# Effects of an early life diet containing large phospholipid-coated lipid globules on hepatic lipid metabolism in mice

**DOI:** 10.1038/s41598-020-72777-y

**Published:** 2020-09-30

**Authors:** Onne A. H. O. Ronda, Bert J. M. van de Heijning, Ingrid Martini, Albert Gerding, Justina C. Wolters, Ydwine T. van der Veen, Martijn Koehorst, Angelika Jurdzinski, Rick Havinga, Eline M. van der Beek, Folkert Kuipers, Henkjan J. Verkade

**Affiliations:** 1 Department of Pediatrics, University Medical Center Groningen, University of Groningen, CA31, PO Box 30001, 9700 RB Groningen, The Netherlands; 2 Danone Nutricia Research, Uppsalalaan 12, 3584CT Utrecht, The Netherlands; 3 Department of Systems Biology, Centre for Energy Metabolism and Ageing, University Medical Center Groningen, University of Groningen, PO Box 30001, 9700RB Groningen, The Netherlands; 4 Laboratory Medicine, University Medical Center Groningen, University of Groningen, PO Box 30001, 9700RB Groningen, The Netherlands

**Keywords:** Metabolism, Nutrition

## Abstract

We recently reported that feeding mice in their early life a diet containing a lipid structure more similar to human milk (eIMF, Nuturis) results in lower body weights and fat mass gain upon high fat feeding in later life, compared to control (cIMF). To understand the underlying mechanisms, we now explored parameters possibly involved in this long-term effect. Male C57BL/6JOlaHsd mice, fed rodent diets containing eIMF or cIMF from postnatal (PN) day 16–42, were sacrificed at PN42. Hepatic proteins were measured using targeted proteomics. Lipids were assessed by LC–MS/MS (acylcarnitines) and GC-FID (fatty-acyl chain profiles). Early life growth and body composition, cytokines, and parameters of bile acid metabolism were similar between the groups. Hepatic concentrations of multiple proteins involved in β-oxidation (+ 17%) the TCA cycle (+ 15%) and mitochondrial antioxidative proteins (+ 28%) were significantly higher in eIMF versus cIMF-fed mice (p < 0.05). Hepatic l-carnitine levels, required for fatty acid uptake into the mitochondria, were higher (+ 33%, p < 0.01) in eIMF-fed mice. The present study indicates that eIMF-fed mice have higher hepatic levels of proteins involved in fatty acid metabolism and oxidation. We speculate that eIMF feeding programs the metabolic handling of dietary lipids.

## Introduction

Milk is an emulsion of fat in water. Its fat droplets are encapsulated by the milk fat globule membrane (MFGM). The MFGM consists of a unique tri-layer phospholipid membrane and envelopes milk fat globules^1,2^. These globules have a mode diameter (the particle diameter most abundant by volume) of approx. 3–5 µm^2–4^. Current infant formulae contain plant-based lipids globules, which are primarily emulsified by proteins, typically do not contain an MFGM^2,5^. The lipid globules in typical infant formulae have a mode diameter of approx. 0.4 µm^2,5^. The physicochemical structure of milk fat globules (the MFGM, i.e. a phospholipid membrane and large diameter), modulates gastrointestinal lipolysis, postprandial lipemia and, to some extent, the postabsorptive metabolism of absorbed fats^6–8^.

Breast milk feeding is epidemiologically associated with a lower incidence of obesity in childhood and adulthood, versus infant milk formula (IMF)-feeding^9^. A distinct compositional and/or physicochemical difference between human milk and formulae have been suggested to underlie these long-term differences. One of the potential drivers for the difference in obesity incidence is thought to be (metabolic) ‘programming’; a stimulus or insult during a sensitive window of development, which has long-term effects on an organism^10,11^. When the physicochemical structure of human milk lipid droplets is mimicked in a rodent diet mixed with an experimental infant milk formula (eIMF) and fed to mice in early life, these mice gain less body weight and fat mass when challenged with a Western style diet later in life compared to a rodent diet mixed with control IMF (cIMF)^12–14^. The eIMF is a concept infant milk formula with large, phospholipid coated lipid droplets (mode diameter 3–5 μm; Nuturis)^2^.

The physicochemical structure of eIMF (large lipid droplets, MFGM-coated) may be responsible for the observed effects on later-life body weight and fat mass gain, for these effects are not found using an IMF containing small MFGM-coated lipid droplets^5^ and neither upon adding MFGM as an ingredient (in free form)^15^. The underlying mechanism of the long-term (programming) effect of eIMF on body weight and fat mass gain has not yet been elucidated. Rapid weight gain in human infancy increases the later-life risk of obesity, type 2 diabetes, the metabolic syndrome and cardiovascular disease^16,17^. A later-life environment which includes overnutrition and physical inactivity (an obesogenic environment) amplifies the aforementioned risk factors^17^. The “Thrifty Phenotype” hypothesis proposes that poor nutrition during early life programs the tissues to more readily store energy whenever available^17^. Adipose tissue is largely responsible for storing that surplus energy, and hence forms a buffer against variations in (lower) dietary intake and (higher) expenditure of energy^18,19^. Beyond its function as an energy storage depot, adipose tissue is recognized as an endocrine entity^17–20^. It plays important roles in the regulation of food intake, energy expenditure and immune function^18^. Adipose tissue mediates these effects through, among others, the secretion of (peptide) hormones such as leptin and adiponectin^17,19,20^. Leptin plays an important neuroendocrine role in metabolic flexibility, defined as the ability to efficiently adapt the metabolism by substrate sensing, trafficking, storage, and utilization, dependent on availability and requirement^21^. Metabolic flexibility is not a binary phenomenon, but involves tightly regulated adjustments mediated by a large array of messengers^21^. Many of these messengers, including insulin, glucagon, and bile acids, show a postprandial response. The postprandial increase in plasma bile acids is known to increase insulin sensitivity and energy expenditure^21,22^. The bile acid-activated nuclear receptor FXR (farnesoid X receptor, NR1H4) is expressed in adipose tissue where it is a determinant of adipose tissue architecture^22^. FXR contributes to whole-body lipid homeostasis^22^. Of interest, in formula-fed piglets, hepatic bile acid synthesis is higher than in breastfed piglets^23^. It is suggested that low dietary cholesterol intake (typical in formula feeding versus breastmilk), or cholesterol bioavailability, stimulates cholesterol synthesis, cholesterol conversion to bile acids, and biliary bile acid secretion^23^. Previously, our group established that the murine intestine can function as an environmental sensor for cholesterol and is able to retain an active metabolic memory for early postnatal cholesterol conditions through epigenetic silencing of the main cholesterol transporter, NPC1L1^24^. It is not yet known which mechanism underlies these observations^24^. Low cholesterol uptake in early life may not only program for altered cholesterol metabolism in later life, but also for altered bile acid metabolism. It remains to be further evaluated whether the bile acid pathways are affected long term through programming mechanisms by postnatal feeding of structured lipids (i.e. eIMF versus cIMF).

Metabolic flexibility is, to some extent, limited by the maximum rate of substrate utilization (the capacity). The liver, adipose tissue, heart and skeletal muscles govern systemic metabolic flexibility^21^. Adipose tissue and skeletal muscle tissue likely play the biggest role^25^. The liver is a central organ in lipogenesis, ketogenesis, gluconeogenesis and glycogenolysis among other metabolic homeostasis functions^21^. Its central role in these processes, and its relatively high metabolic fluxes and resting metabolic rate in men^26,27^ and mice^27^, make it an interesting organ to study in terms of metabolic flexibility and rate of oxidation^21,28^. Mitochondria play a crucial role in determining the maximal substrate utilization rate and therefore determine, to some extent, metabolic flexibility^21^. Herein, the exercise-activated transcriptional co-activator PGC1α (PPAR gamma coactivator 1-alpha) responds, together with the appropriate transcription factors, such as PPARG and its RXR heterodimer, to increased AMP/ATP ratios via AMPK^29^. PGC1α is involved in the regulation of expression of genes involved in mitochondrial energy homeostasis and metabolic adaptations^28^, including nuclear respiratory factors (NRFs) and Peroxisome proliferator-activated receptors (PPARs). NRFs and PPARs regulate the expression of nuclear genes involved in oxidative phosphorylation, substrate transportation and fatty acid oxidation^21,30^.

We aim to get a better understanding of the underlying mechanisms of eIMF-induced early life programming with regards to its long-term effects on body weight and fat mass gain. We determined the possible involvement of a set of relevant metabolic parameters in the long-term effect of eIMF on body weight and fat mass gain. We compared eIMF and cIMF-fed mice with respect to early life growth rate and body composition, plasma adipokines and cytokines and parameters of bile acid metabolism. To assess lipid metabolism, we assessed (adipose) tissue weights, hepatic markers of mitochondrial substrate utilization, plasma lipid profiles, fatty acyl chain profiles and relevant gene expression patterns.

## Results

### Early life growth, plasma lipids and adipokines

**Figure 1:**
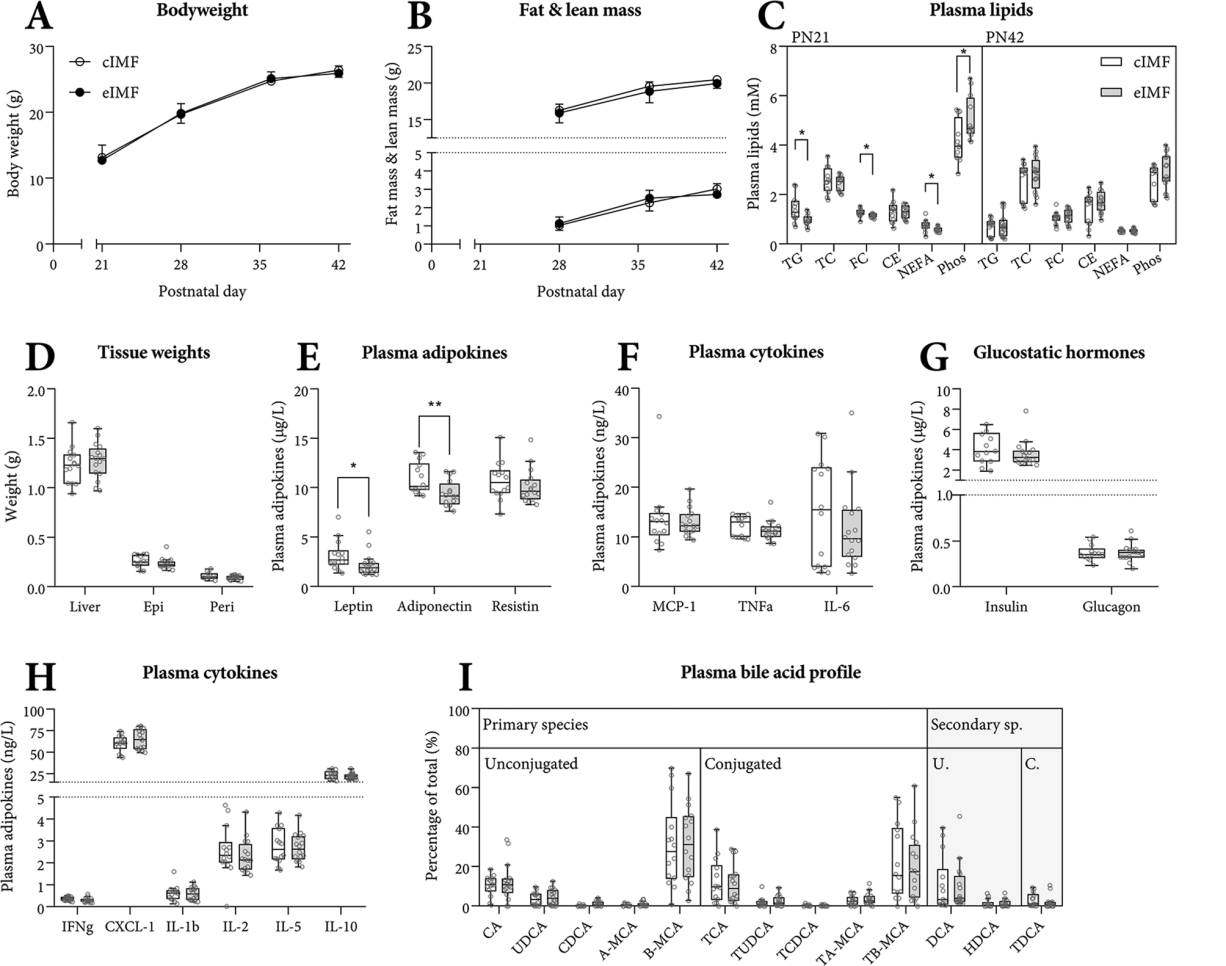
Body weight, fat mass and lean mass gain during eIMF or cIMF feeding. Plasma lipids, adipokines, cytokines and bile acids at PN42. Body weight (**A**), fat and lean mass (**B**) are expressed in absolute weights. Plasma lipids at PN21 (weaning) and at PN42 (**C**). All other parameters were measured at PN42. Liver, epididymal (epi) and perirenal (peri) fat pads weights obtained at dissection at PN42 are expressed absolutely (**D**). Plasma adipokines (**E**,**F**), glucostatic hormones (**G**) and cytokines (**H**, all 4 h fasting at PN42) are expressed as absolute concentration. Plasma bile acid species are expressed as a percentage (**I**, PN42). *TG* triglycerides, *TC* total cholesterol, *FC* free cholesterol, *CE* cholesterol ester, *NEFA* non-esterified fatty acids, *Phos* phospholipids, *MCP-1* monocyte chemoattractant protein-1, *TNFα* tumor necrosis factor alpha, *IL-1b/2/5/6/10* interleukin 1b/2/5/6/10, *IFNg* interferon gamma, *CXCL-1* CXC chemokine ligand 1, *(T-) (L) CA* (tauro-) (litho) cholic acid, *(T/G-) (U/C/H) DCA* (tauro-) (urso/cheno/hyo) deoxycholic acid, *(T)A/B-MCA* (tauro-) α/β-muricholic acid. (**A**–**I**) n = 12–16; (**A**,**B**) Median ± interquartile range. (**C**–**I**) Tukey boxplots and scatter plots. Exact two-sided Mann–Whitney *U* test **p < 0.01, *p < 0.05.

Body weight (Fig. 1A), fat mass and lean mass (Fig. 1B) gain, and tissue weights (Fig. 1D) were similar between groups. At weaning (PN21), after 5 days of IMF feeding, unfasted plasma triglycerides, cholesterol and NEFA were subtly lower, whereas phospholipids were higher in eIMF- versus cIMF-fed mice (Fig. 1C). At PN42, after a 4 h fast, plasma lipids were similar between groups, though adipokines leptin and adiponectin were lower in eIMF-fed mice (Fig. 1E). The calculated average (SD) leptin/adiponectin ratio was 0.24 (0.13) versus 0.30 (0.17) for eIMF and cIMF, respectively (NS). Other adipokines and cytokines (resistin, MCP-1, TNFα, IL-6, Fig. 1E,F), glucostatic hormones (Fig. 1G) and cytokines (Fig. 1H) were similar between groups. The plasma bile acid profiles were similar between IMF groups at PN21 (data not shown) and at PN42 (Fig. 1I), suggesting similar luminal bile acid composition in terms of hydrophobicity.

### Markers of fatty acid oxidation

**Figure 2:**
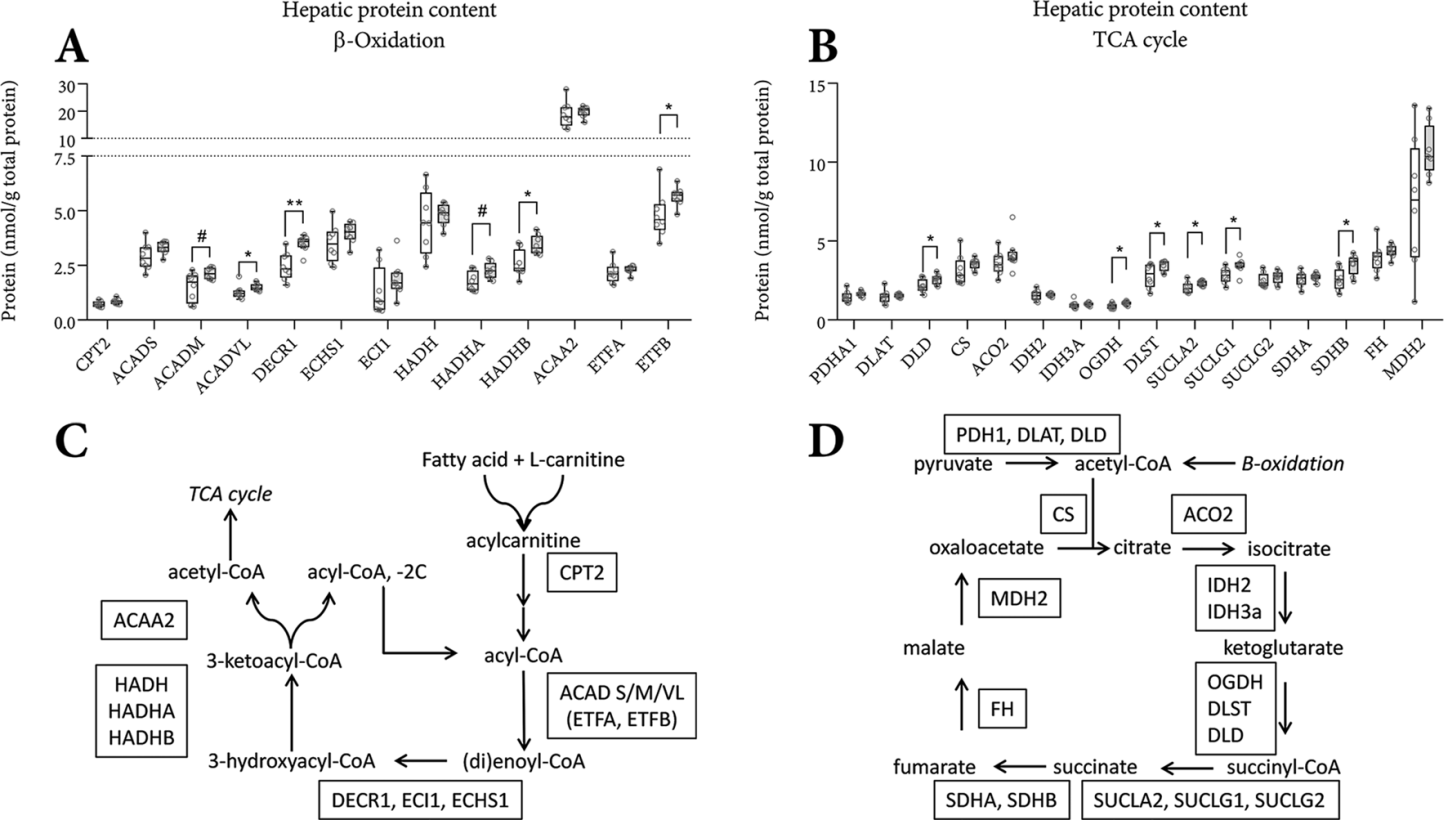
Hepatic levels of proteins involved in β-oxidation and the tricarboxylic acid (TCA) cycle at PN42. Mitochondrial proteins involved in fatty acid β-oxidation (**A**), and the TCA cycle (**B**), were quantified in whole liver homogenates by targeted proteomics^50^ using isotopically (^13^C-) labeled standards derived from synthetic peptide concatemers (QconCAT) using mass spectrometry technology. Schematic representation of β-oxidation (**C**), and the TCA cycle (**D**) with the quantified targets placed at their respective position for the purpose of clarification. All values are expressed as nanomole per gram total protein. (**A**,**B**) n = 8; Tukey boxplots and scatter plots. Exact two-sided Mann–Whitney *U* test **p < 0.01, *p < 0.05, ^#^p < 0.1.

**Figure 3:**
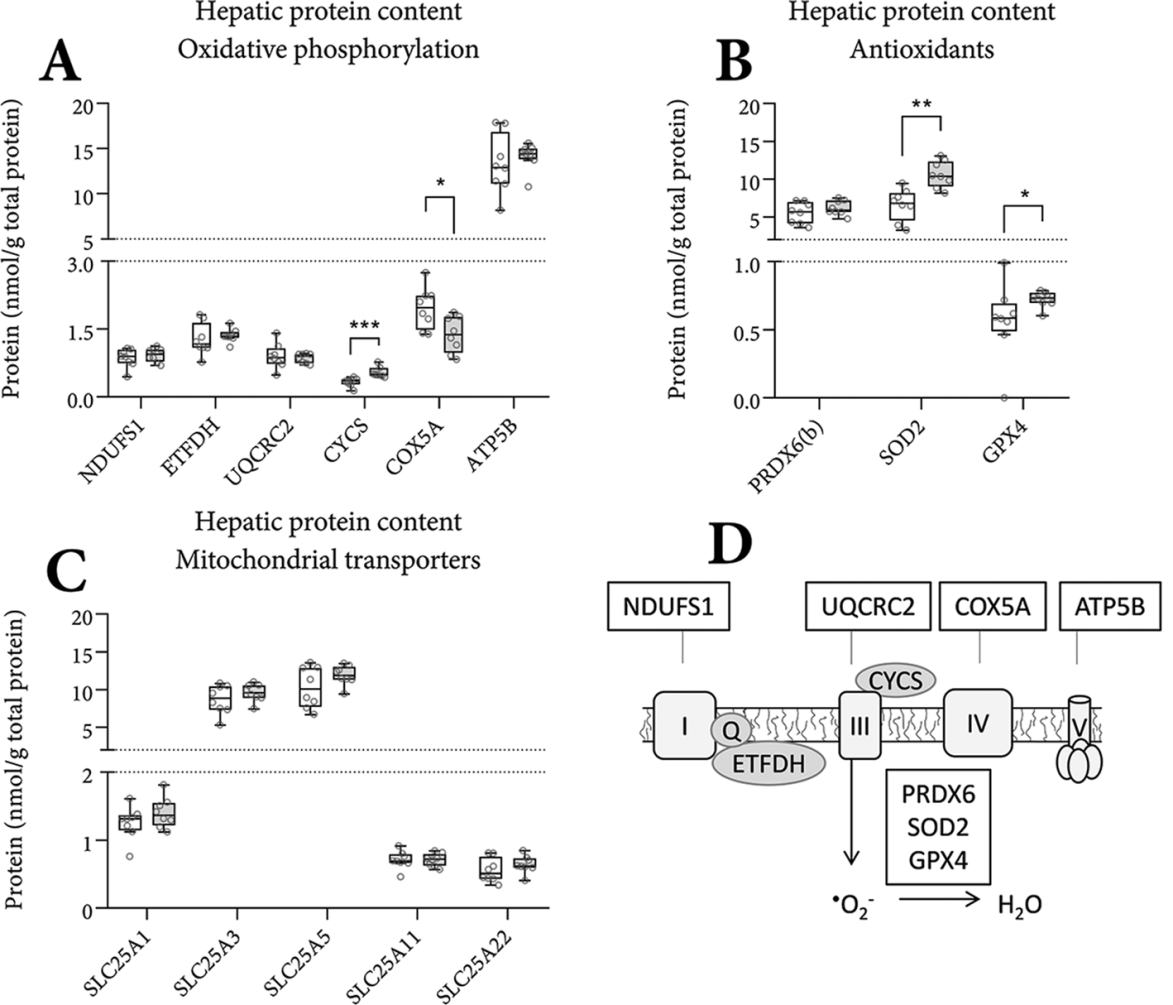
Hepatic levels of proteins involved in oxidative phosphorylation, antioxidation and substrate transport at PN42. Mitochondrial proteins involved in oxidative phosphorylation (**A**), antioxidation (**B**), and substrate transport (**C**) were quantified in whole liver homogenates by targeted proteomics^50^ using isotopically (^13^C-) labeled standards derived from synthetic peptide concatemers (QconCAT) using mass spectrometry technology. Schematic representation of oxidative phosphorylation and mitochondrial antioxidation with the quantified targets placed at their respective positions (D) for the purpose of clarification. All values are expressed as nanomole per g total protein. (**A**–**C**) n = 8; Tukey boxplots and scatter plots. Exact two-sided Mann–Whitney *U* test ***p < 0.001, **p < 0.01, *p < 0.05.

Using mass spectrometry technology, we quantified hepatic concentrations of mitochondrial proteins involved in β-oxidation (Fig. 2A), the TCA cycle (Fig. 2B), electron transport (Fig. 3A), antioxidative proteins (Fig. 3B), and substrate transportation (Fig. 3C). Fatty acids, once transported into the mitochondrion’s matrix via the carnitine shuttle, are successively chain-shortened via the β-oxidation cycle (Fig. 2A,C). We noted higher protein concentrations of ACADVL, DECR1, HADHB, and ETFB in eIMF-fed mice, suggesting a higher β-oxidation capacity. ACADM and HADHA were non-significantly higher in eIMF-fed mice. Each β-oxidation cycle results in an acetyl-CoA and a 2C-shortened acyl-CoA. The acetyl-CoA can enter the TCA cycle. During the β-oxidation cycle, several electron carriers are reduced (that is, they accept electrons), which are oxidized by the electron transport chain. The tricarboxylic acid (TCA) cycle (Fig. 2B,D) oxidizes acetyl-CoA derived from a variety of sources, including glycolysis and the aforementioned β-oxidation. Protein concentrations of DLD, OGDH, DLST, SUCLA2, SUCLG1 and SDHB were higher in eIMF-fed mice, suggesting a higher TCA cycle capacity. Each TCA cycle reduces several electron carriers, which are used by the electron transport chain.

### Markers of oxidative phosphorylation

Oxidative phosphorylation (Fig. 3A,D) encompasses the last step in substrate oxidation towards ATP production. Herein, energy from the chemical bonds in fatty acids and carbohydrates, carried by electron carrier molecules, is used to create a proton gradient across the inner mitochondrial membrane. Protons are obtained from water molecules, whereby oxygen radicals are generated. The free radicals are oxidized back to water by various peroxidase and dismutase enzymes. The proton gradient is finally used to synthesize ATP from ADP and inorganic phosphate. We noted a higher protein concentrations of cytochrome *c* (CYCS), though a lower concentration of COX5A in eIMF-fed mice (Fig. 3A). Antioxidant enzymes SOD2 and GPX4 were higher in eIMF-fed mice (Fig. 3B). Hepatic mitochondrial substrate carriers (Fig. 3C) SLC25A1, A3, A5, A11 and A22 had similar protein levels between groups.

### Hepatic l-carnitine and acylcarnitine species

Higher tissue levels of l-carnitine are expected upon higher β-oxidation rate, to allow for fatty acid transportation across the mitochondrial membrane. Therefore, we measured hepatic free and bound carnitine species (Table 1). The cIMF and eIMF diets contained similar l-carnitine levels (32 ng/g and 37 ng/g respectively, both within the EU legal margins for infant formulae). Yet, hepatic free l-carnitine levels were significantly higher (+ 33%, p < 0.01) in eIMF-fed mice. Bound acylcarnitine species (C2–C18) were comparable between groups. Of note, the sum of hydroxybutyrylcarnitine and malonylcarnitine, which are analytically indistinguishable, was higher (+ 27%, p < 0.05). Mainly as a result of higher free l-carnitine, the free to bound carnitine ratio was higher (+ 56%, p < 0.01) in eIMF-fed mice.

**Table 1:** Hepatic l-carnitine and acylcarnitine species at PN42.

	Liver (nmol/g)				P-value	
	cIMF		eIMF			
	Median	IQR	Median	IQR		
Sum	184	35	214	51	< 0.05	
Sum C14–C18	0.40	2	0.27	1	n.s.	
Free/bound ratio	3.4	2	5.2	3	< 0.01	

Common name	Abbreviation	Liver (nmol/g)				P-value
		cIMF		eIMF		
		Median	IQR	Median	IQR	
l-Carnitine	C0	134	30	172	53	< 0.01
Acetylcarnitine	C2	9.9	16	6.4	7	n.s.
Propionylcarnitine	C3	0.77	0.8	0.90	0.6	n.s.
Butyrylcarnitine	C4	0.20	0.5	0.13	0.3	n.s.
Tiglylcarnitine	C5:1	*Trace*				–
Isovaleryl carnitine	C5	*Trace*				–
Hexanoylcarnitine	C6	*Trace*				–
Octanoylcarnitine	C8	*Trace*				–
Decenoylcarnitine	C10:1	*Trace*				–
Decanoylcarnitine	C10	*Trace*				–
Dodecenoylcarnitine	C12:1	0.33	0.1	0.27	0.08	n.s.
Dodecanoylcarnitine	C12	*Trace*				–
Tetradecenoylcarnitine	C14:1	*Trace*				–
Tetradecanoylcarnitine	C14	*Trace*				–
Hexadecenoylcarnitine	C16:1	*Trace*				–
Hexadecanoylcarnitine	C16	*Trace*				–
Octadecadienoylcarnitine	C18:2	*Trace*				–
Octadecenoylcarnitine	C18:1	0.10	1.0	0.07	0.7	n.s.
Octadecanoylcarnitine	C18	*Trace*				–
Butyrylcarnitine + Malonylcarnitine	C4OH + C3DC	3.1	0.7	4.1	1.6	< 0.05
3-OH-isovalerylcarnitine + Methylmalonylcarnitine	C5OH + C4DC	0.60	0.2	0.70	0.2	n.s.
Glutarylcarnitine	C5DC	11	12	10	7	n.s.
3-Methylglutarylcarnitine	C6DC	0.87	1.5	0.73	0.3	n.s.
3-OH-Dodecanoylcarnitine	C12OH	6.9	2.9	6.0	2	n.s.

Liver acylcarnitine species (in nmol/g) represent median and interquartile range (IQR). *Trace*: Near or below lower limit of quantification. A ‘+’ symbol indicates the sum of 2 analytically indistinguishable compounds. Exact two-sided Mann–Whitney *U* test.

*n.s.* not significant.

### Hepatic mRNA expression markers of lipid metabolism

**Figure 4:**
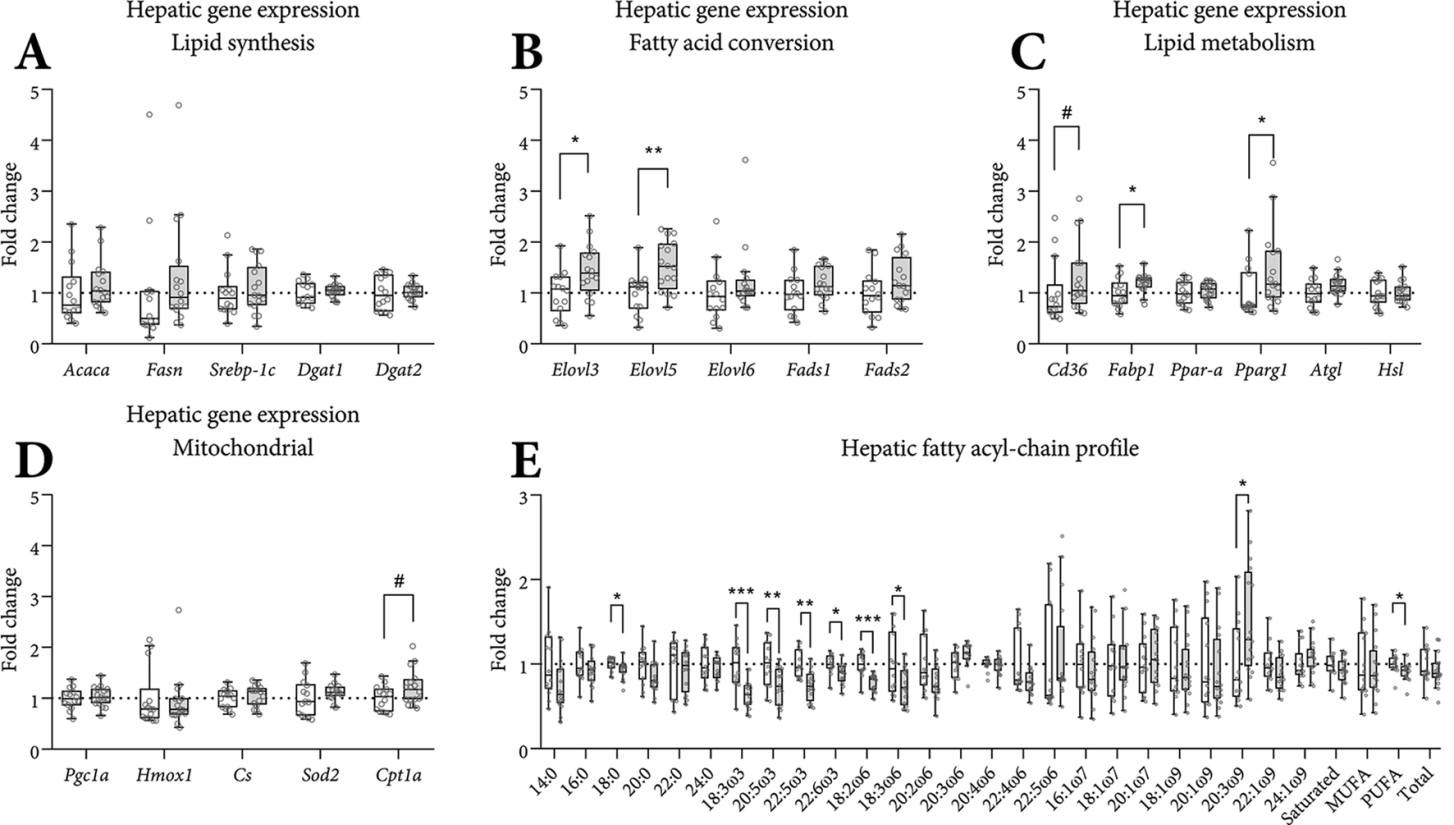
Hepatic mRNA gene expression of lipid metabolism genes at PN42. Gene expression patterns, normalized to *36b4* and shown as fold-change versus cIMF, for lipid synthesis (**A**), fatty acid conversion enzymes (**B**), lipid metabolism (**C**) and mitochondria (**D**). The hepatic fatty acyl chain profile (**E**) is shown as fold-change versus cIMF. See Table 2 for fatty acyl-chain abbreviations. *Saturated* Σ saturated moieties, *MUFA* Σ mono-unsaturated moieties, *PUFA* Σ poly-unsaturated moieties, *total* Σ all assessed fatty moieties. (**A**–**E**) n = 14–16; Tukey boxplots and scatter plots. Exact two-sided Mann–Whitney *U* test ***p < 0.001, **p < 0.01, *p < 0.05, ^#^p < 0.1.

To further characterize the short-term (i.e. 26 days) effect of eIMF-feeding on lipid synthesis (Fig. 4A), fatty acid species conversion (Fig. 4B), lipid metabolism (Fig. 4C) and mitochondrial targets (Fig. 4D), we performed qPCR analyses and determined the hepatic fatty acyl-chain profile (Fig. 4E). Hepatic expression of genes related to lipid synthesis (Fig. 4A; *Acaca*, *Fasn*, *Srebp-1c*, *Dgat1*, *Dgat2*) was similar between groups. Expression of fatty acid elongation genes (Fig. 4B) *Elovl3* (+ 44%) and *Elovl5* (+ 53%) was higher in eIMF-fed mice. Expression of *Elovl6*, and genes involved in fatty acid desaturation (*Fads1* and *Fads2*), were similar between groups. Expression of the fatty acid transporter *Cd36* was non-significantly higher (Fig. 4C, + 31%, p = 0.06), and the liver-type fatty acid binding protein *Fabp1* was higher (+ 20%). *Ppar-a* was similar between groups. Expression of *Pparg* (+ 45%) was higher in eIMF. Expression of the triacylglycerol lipase *Atgl* and the diacylglycerol lipase *Hsl* were similar between groups. Mitochondrial biogenesis is regulated by *Pgc1a* (Fig. 4D), which had similar expression levels between groups. In addition, its downstream target, *Hmox1* and *Fasn*, were similar. Expression levels of citrate synthase (*Cs*) and *Sod2* were similar between groups. This suggests that under these conditions the effect on SOD2 protein levels is regulated beyond transcription. *Cpt1a* expression was non-significantly higher (+ 21%, p = 0.08) in eIMF-fed mice. Mice fed eIMF had a subtly different hepatic fatty acyl-chain profile (Fig. 4E), whereas the diets had a similar composition (Table 2). Of note, the hepatic presence of the dominant dietary ω-3 and ω-6 moiety (18:3ω3 and 18:2ω6) was lower (− 36% and − 22% respectively, p < 0.001) in eIMF-fed mice. Derivative ω-3 fatty acids (20:5ω3, 22:5ω3 and 22:6ω3) were lower in eIMF-fed mice, whereas derivative ω-6 fatty acids were similar. The sum of the assessed fatty moieties was similar between groups.

**Table 2:** Fatty acid composition of the diets.

Common name	Abbreviation	Control IMF	Experimental IMF
**Σ Saturated moieties**		44	42
Myristic acid	14:0	8.9	7.1
Palmitic acid	16:0	26	25
Stearic acid	18:0	7.8	8.9
Arachidic acid	20:0	0.28	0.32
Behenic acid	22:0	0.27	0.39
Lignoceric acid	24:0	0.17	0.26
Cerotic acid	26:0	0.032	0.039
**Σ Monounsaturated moieties**		36	39
Palmitoleic acid	16:1ω7	1.2	1.1
Vaccenic acid	18:1ω7	1.9	1.9
Oleic acid	18:1ω9	33	35
Gondoic acid	20:1ω9	0.38	0.42
Erucic acid	22:1ω9	0.080	0.13
Nervonic acid	24:1ω9	0.053	0.074
**Σ Polyunsaturated moieties**		20	19
	Σ ω-3 species	3.4	3.4
α-Linolenic acid	18:3ω3	2.8	2.8
Eicosapentaenoic acid	20:5ω3	0.12	0.12
Docosapentaenoic acid	22:5ω3	0.090	0.099
Docosahexaenoic acid	22:6ω3	0.38	0.38
	Σ ω-6 species	16	16
Linoleic acid	18:2ω6	16	15
γ-linolenic acid	18:3ω6	0.050	*-*
Eicosadienoic acid	20:2ω6	0.046	*-*
Dihomo-γ-linolenic acid	20:3ω6	0.091	0.12
Arachidonic acid	20:4ω6	0.44	0.43
	Σ ω-6/Σ ω-3 ratio	4.8	4.7
Mead acid	20:3ω9	0.38	0.42

Fatty acid composition (FA weight%) of the diets (given during postnatal day 16–42), as measured by fatty acyl chain profiling.

## Discussion

In the present study, we show that growth rates and body composition were similar during early life eIMF versus cIMF feeding. At PN42, corresponding with 26 days of either eIMF or cIMF feeding, plasma cytokines and bile acid metabolism were similar between groups. These data render it unlikely that the long-term effects of eIMF on body weight and fat mass gain following a Western-style diet^5,14^ are mechanistically related to these parameters. At the end of the IMF feeding period, prior to a (high fat) dietary challenge, we do see significantly higher levels of hepatic proteins involved in fatty acid oxidation and the TCA cycle. Our data suggest that the hepatic activity of the TCA cycle, i.e. the average rate at which lipids and/or carbohydrates are being oxidized, is higher after early life eIMF feeding in mice.

Our data show higher levels of a range of hepatic proteins (and enzymes) involved in β-oxidation, TCA cycle, antioxidative enzymes, together with higher carnitine levels, and a higher hepatic expression of *Fabp1*. These observations led us to speculate that hepatic β-oxidation rate (or at least, its capacity) is higher in eIMF-fed mice (Figs. 2,3). The higher hepatic protein levels of the mitochondrial antioxidants SOD2 and GPX4 possibly indicate a higher net capacity to remove reactive oxygen species. The antioxidant protein superoxide dismutase 1 (SOD1) and cytosolic catalase T (CTT1) also play major roles in the removal of superoxide anion radicals and H_2_O_2_ in the liver^31,32^. As electron chain complexes I and III produce oxygen radicals (i.e. reactive oxygen species) upon proton pumping, we speculate that the higher SOD2 and GPX4 levels are the result of a higher ATP synthesis rate^33–36^. Hepatic protein levels of citrate synthase (CS), of which the activity is considered the rate-limiting step in the TCA cycle^37^, were similar between our groups (Fig. 2B). Yet, CS activity can vary with similar CS protein levels, such as when comparing a resting state to an active state^37^. The mitochondrial protein levels and the concomitantly higher l-carnitine levels are suggestive, but not conclusive, of an average inter-day higher rate of oxidation.

The TCA cycle can dissipate acetyl-CoA derived from the catabolism of lipids, carbohydrates, certain amino acids and minor miscellaneous sources (e.g. ethanol, ketone bodies). Higher protein levels of TCA cycle enzymes may correspond with a higher TCA cycle flux. The concomitant higher levels of proteins involved in β-oxidation^38^ and the higher hepatic carnitine concentration are suggestive of higher β-oxidation activity^39,40^. An increased flux through the β-oxidation cycle, also yielding more acetyl-CoA, would then require a higher flux through the TCA cycle. Carnitine is required for FA transportation into the mitochondrial matrix, and thus for an efficient fatty acid oxidation (Table 1). Hepatic carnitine can be obtained from the diet, can be locally biosynthesized, or can originate from tissue redistribution. Carnitine biosynthesis is higher in conditions in which rates of β-oxidation are chronically elevated^39,40^. Negligible accumulation of (long-chain) acylcarnitine species, seen in the livers of both cIMF and eIMF-fed mice, implies that β-oxidation does not outpace the TCA cycle in this tissue^29,41^. This implication is backed up by the simultaneously higher levels of proteins involved in β-oxidation and proteins involved in the TCA cycle, and the higher fractional and absolute levels of l-carnitine. These observations suggest a more active fat metabolism and oxidation, but they are not conclusive. In vivo measurements of mitochondrial substrate utilization, using (for instance) isotopically-labelled lipid tracers, would be helpful to quantitate rates of oxidation. This type of experiments was beyond the scope of the present study, but, to our opinion, would be worthwhile as subsequent next step. Recently, we found that early life eIMF feeding (versus cIMF) programs later life postabsorptive lipid trafficking in high-fat diet but not in low-fat diet fed mice^42^. It is not yet known whether the effects on mitochondrial protein levels (this study and Kodde et al*.*^43^) and the effects on postabsorptive lipid trafficking are mechanistically related.

The observations on lower leptin and adiponectin levels in mice fed eIMF (Fig. 1E) may offer insights into the mechanism behind the differences found in body weight and composition in later life^11,20^. Leptin is involved in the regulation of food intake and is primarily synthesized and secreted by adipose tissue, in proportion to the amount of body fat^19^. Adiponectin is a classic anti-inflammatory agent and is known to enhance fatty acid oxidation^19^. Plasma adiponectin is lower in obese subjects^19^, so is thought to negatively correlate with the amount of body fat^44^. We are unsure how to interpret our data, which discrepantly show lower plasma leptin and lower adiponectin levels, and a similar leptin-to-adiponectin ratio (Fig. 1E). In human subjects, plasma adiponectin levels can be decreased by unprocessed diets versus ultra-processed diets^45^. Possibly, ultra-processed diets are more similar to cIMF than to eIMF, with regards to the physicochemical structure . After all, the eIMF, compared to cIMF, more closely resembled breastmilk with regards to physicochemical structure^2^. It is not well understood how plasma adiponectin levels are regulated (if at all) by food intake, composition or physicochemical structure. Though, our data do suggest that plasma adiponectin levels are also regulated by means other than by the amount of body fat^19,44^.

Previously, it was noted that, upon a Western style diet challenge, gene and protein expression of mitochondrial oxidative capacity markers were higher in skeletal muscle and adipose tissue in eIMF compared to cIMF-primed mice^43^. It is not yet known whether the observed effects on hepatic β-oxidation proteins persist into adulthood. We speculate that this early life phenomenon may be a potential trigger for, or consequence of, metabolic programming, and is mechanistically involved in the observed long-term effect on body weight and fat mass gain.

## Methods

### Animals and study design

Experimental procedures were approved by an external independent animal experiment committee (CCD, Central Animal Experiments Committee, The Netherlands), and after positive advice by the Committee for Animal Experimentation of the University of Groningen. Subsequently, the study design was approved by the local Animal Welfare Body Procedures complied with the principles of good laboratory animal care following the European Directive 2010/63/EU for the use of animals for scientific purposes. All animals were kept in a temperature-controlled room (21 ± 1 °C, 55 ± 10% humidity, lights on 8 AM–8 PM) in type 1L (360 cm^2^) polysulfone cages bearing stainless-steel wire covers (UNO BV, the Netherlands), with wood shaving bedding, Enviro-dri (TecniLab, The Netherlands) and cardboard rolls. All mice were handled by the same researcher (OR). Virgin C57BL/6JOlaHsd breeders (11M, 22F) 12 weeks of age (Envigo, The Netherlands) were acclimatized for 2 weeks. They were time-mated in 2F + 1M couples. See^12^ for the paradigm used. Males were removed after 2 days. Pregnancy was confirmed by a > 2 g increase in body weight after 1 week. Pregnancy occurred in 15 females. Nonpregnant females were mated again for a maximum of 4 times. Delivery day was recorded as postnatal day (PN) 0. Pups were randomized between dams, and litters were culled to 4M + 2F at PN2, weaned at PN21, and diets were provided as daily freshly prepared dough balls (40% water) from PN16 to PN42^12,13^. Care was taken to minimize handling and stress prior to weaning. No measurements were made prior to weaning. It was reasoned that handling prior to weaning may have disturbed the IMF’s metabolic programming potential and/or the stress of handling may have programmed the pups in itself. It was assumed that any differences measured at PN21 were due to the IMF. This study was not performed blinded as the programming diets were visually distinct. Breeders and female offspring were terminated (CO_2_) at weaning, in compliance with the AVMA Guidelines for the Euthanasia of Animals.

### Programming diets

Two IMF powders (Nutricia Cuijk B.V., Cuijk, the Netherlands) were used. The IMF powders had a similar macro- and micronutrient content (Table 3), as provided by the supplier. The lipid moieties of the two IMF powders both comprised about 50% vegetable oil and 50% milkfat and had a similar fatty acid profile (Table 2), as assessed internally with methodology shown below. The cIMF comprised fat globules with a volume moment mean (De Brouckere Mean Diameter; D[4,3]) of 0.8 µm and a mode diameter 0.5 µm. The eIMF comprised phospholipid-coated (Lipamin M 20, Lecico, France) lipid globules with a D[4,3]) of 7 µm, and a mode diameter 4.2 µm, explained in more detail elsewhere^2^. The eIMF (Nuturis) is defined as a concept infant milk formula with large, phospholipid coated lipid droplets with mode diameter 3–5 μm^2^. IMF powders (283 g/kg feed) were supplemented with protein and carbohydrate (Ssniff Spezialdiäten GmbH, Soest, Germany) to obtain AIN-93G-compliant diets, with a fat moiety (~ 7 w%) derived entirely from IMF^46^.

**Table 3:** Calculated nutrient composition of the diets.

	Control IMF	Experimental IMF		
**Carbohydrate**	609	618		
Mono/di-saccharides	225	235		
Glucose	3.7	3.4		
Lactose	134	144		
Sucrose	85	85		
Other sugars	2.6	2.4		
Polysaccharides	380	380		
Maltodextrin	101	101		
Corn starch	280	280		
Other	0.84	0.68		
**Fiber**	49.0	48.2		
Cellulose	32.0	32.0		
Fructo-oligosaccharides	1.7	1.4		
Galacto-oligosaccharides	15.3	14.3		
**Lipids**	77.2	70.6		
Vegetable fat	37.5	32.9		
Milkfat	38.6	36.7		
Other animal fat	1.1	0.98		
Phospholipids	0.084	1.1		
Cholesterol	0.12	0.12		
**Protein**	199	198		
Whey	17.6	16.5		
Casein	181	181		

Particle size	Mean	SD	Mean	SD
Mode diameter (µm)	0.5	0.08	4.2	0.9
D [4,3] (µm)	0.81	0.2	6.8	0.2
D [3,2] (µm)	0.43	0.004	0.86	0.1
Surface area (m^2^/g)	15	0.2	7.7	1.0
Total energy, kcal/g (kJ/g)	3.87 (16.2)		3.87 (16.2)	

Calculated nutrient composition (in g/kg) of the diets (given during postnatal day 16–42).

### Body composition

Lean and fat mass was quantified by time-domain nuclear magnetic resonance (LF90II, Bruker Optics, Billerica, MA, USA), not requiring fasting or anesthesia as described elsewhere^14^. Measurements were done in the same animals at PN28, PN35 and PN41.

### Termination

Mice were anaesthetized (isoflurane/O_2_) after a 4-h fasting period (during light phase; 9 AM–1 PM) and sacrificed by heart puncture; a terminal blood sample was drawn.

### Assays

Plasma was analyzed using the V-PLEX Proinflammatory Panel 1 (mouse) kit (K15048D), Mouse Adiponectin Kit (K152BXC), Mouse Leptin Kit (K152BYC), Mouse MCP-1 Ultra-Sensitive Kit (K152AYC), Mouse/Rat Total Active GLP-1, Insulin, Glucagon Kit (K15171C) and the Mouse/Rat Resistin Kit (K152FNC). Analyses were performed according to the manufacturer’s instructions (Meso Scale Diagnostics LLC, USA). Plasma was analyzed using commercially available kits for triglycerides (Roche, 11877771216), total cholesterol (Roche, 11491458216), free cholesterol (Spinreact, 41035), NEFA (Sopachem, 157819910935), and phospholipids (Sopachem, 157419910930). Esterified cholesterol was calculated as the difference between total and free.

### Plasma bile acids

Using liquid chromatography-mass spectrometry, plasma bile acid species were quantified^47^. To 25 µl of plasma, we added a mixture of internal standards (isotopically labelled bile acids). Samples were centrifuged at 15,800×*g* and the supernatants were transferred and evaporated at 40 °C under a stream of N_2_. Samples were reconstituted in 200 µl methanol:water (1:1), mixed and centrifuged at 1800×*g* for 3 min. The supernatant was filtered using a 0.2 µm spin-filter at 2000×*g* for 10 min. Filtrates were transferred to vials and 10 µl was injected into the LC–MS system. The LC–MS system consisted of a Nexera X2 Ultra High Performance Liquid Chromatography system (SHIMADZU, Kyoto, Japan), coupled to a Sciex Qtrap 4500 MD triple quadrupole mass spectrometer (SCIEX, Framingham, MA, USA). Data were analyzed with Analyst MD 1.6.2 software.

### Fatty-acyl chain profiling

Fatty acid methyl esters (FAMEs) were quantified using gas chromatography^14,48^. Cryogenically crushed tissues were homogenized in Potter–Elvehjem tubes in ice-cold phosphate buffered saline (PBS) solution. A known quantity of homogenized tissue, plasma or food was transferred to glass tubes, and capped with silicone-ptfe septum screw caps. An internal standard (heptadecanoic acid, C17, Sigma, St. Louis, MO, USA) was added. Lipids were trans-methylated at 90 °C for 4 h in 6 M HCl:methanol (ratio 1:5), liquid–liquid extracted twice using hexane, transferred to a clean tube, dried at 45 °C under a stream of N_2_, reconstituted in hexane and transferred to GC vials with inserts. Samples were analyzed by gas chromatography as previously described^48^. The GC system consisted of 6890N network gas chromatograph (Agilent) and was equipped with a HP- ULTRA 1 (50 m length × 0.2 mm diameter, 0.11 µm film thickness) column.

### Acylcarnitine profiling

Acylcarnitine species were quantified using liquid chromatography-tandem mass spectrometry^49^. To 50 µl liver homogenate, prepared as described above, a mixture of internal standards (isotopically labelled acylcarnitine species) and acetonitrile was added. Samples were mixed and centrifuged (15,000×*g*) to precipitate proteins. Supernatant was transferred to GC vials. Samples were analyzed using LC–MS/MS as previously described^49^. The LC–MS/MS system consisted of an API 3000 LC–MS/MS equipped with a Turbo ion spray source (Applied Biosystems/MDS Sciex, Ontario, Canada). Data were analyzed with Analyst and Chemoview software (Applied Biosystems/MSDSciex).

### Targeted proteomics

Targeted quantitative proteomics was performed on mitochondrial targets involved in substrate transport, fatty acid oxidation, the tricarboxylic acid (TCA) cycle, and the detoxification of reactive oxygen species. We used isotopically labeled concatemers as internal standards designed to target murine mitochondrial proteins. The internal standards were derived from synthetic peptides (PolyQuant GmbH, Bad Abbach, Germany) developed as previously described^50^. The method relies on targeted LC–MS/MS in the selected reaction monitoring (SRM) mode to quantify 55 murine mitochondrial proteins in a single run^50^. This method was optimized in isolated mitochondrial fractions from mouse and rat liver and cultured human fibroblasts and in total liver extracts from mice, rats, and humans. In this study, we used total liver extracts. The targeted proteomics approach is suitable and validated for the quantification of proteins in the mitochondrial energy metabolic pathways in mouse, rat, and human samples^50–52^. In our proteomics approach, the exact amino acid sequence of the peptides is known, and *bona fide* reference samples were available to test the performance of the assay in the context of the particular experiment. Targeted LC–MS/MS proteomics is a powerful and superior alternative for immune-based quantitative techniques, when internal controls are available^50,53^.

### Gene expression

Quantification of gene expression was performed as previously described^24^. Using TRI-Reagent (Sigma, St. Louis, MO), total RNA was extracted from cryogenically crushed whole livers. RNA was quantified by NanoDrop (NanoDrop Technologies, Wilmington, DE, USA). Integrity was confirmed by observing ribosomal bands on 1% agarose in TAE. cDNA was synthesized using M-MLV (Invitrogen, Breda, the Netherlands) and random nonamers (Sigma). cDNA was quantified by relative standard curves using quantitative real-time PCR as previously described^24^. Primer and TaqMan probe sequences are given in Table 4.

**Table 4:** Primer and TaqMan probe sequences.

Gene	NCBI RefSeq identifier	Forward primer 5′ → 3′	Reverse primer 5′ → 3′	TaqMan probe 5′ → 3′
*36b4*	NM_007475	GCTTCATTGTGGGAGCAGACA	CATGGTGTTCTTGCCCATCAG	TCCAAGCAGATGCAGCAGATCCGC
*Acaca*	NM_133360.2	CCATCCAAACAGAGGGAACATC	CTACATGAGTCATGCCATAGTGGTT	ACGCTAAACAGAATGTCCTTTGCCTCCAAC
*Cd36*	BC010262	GATCGGAACTGTGGGCTCAT	GGTTCCTTCTTCAAGGACAACTTC	AGAATGCCTCCAAACACAGCCAGGAC
*Cpt1a*	NM_013495.1	CTCAGTGGGAGCGACTCTTCA	GGCCTCTGTGGTACACGACAA	CCTGGGGAGGAGACAGACACCATCCAAC
*Cs*	NM_026444.3	AAGACGTGTCAGATGAGAAGTTACGA	TCCTCAGTACTGCATGACCGTATC	CTCAATTCAGGACGGGTGGTCCCA
*Dgat1*	NM_010046.2	GGTGCCCTGACAGAGCAGAT	CAGTAAGGCCACAGCTGCTG	CTGCTGCTACATGTGGTTAACCTGGCCA
*Dgat2*	NM_026384.2	GGGTCCAGAAGAAGTTCCAGAAG	CCCAGGTGTCAGAGGAGAAGAG	CCCCTGCATCTTCCATGGCCG
*Elovl5*	NM_134255.2	TGGCTGTTCTTCCAGATTGGA	CCCTTTCTTGTTGTAAGTCTGAATGTA	CATGATTTCCCTGATTGCTCTCTTCACAAAC
*Elovl6*	NM_130450.2	ACACGTAGCGACTCCGAAGAT	AGCGCAGAAAACAGGAAAGACT	TTTCCTGCATCCATTGGATGGCTTC
*Fabp1*	NM_017399	GAACTTCTCCGGCAAGTACCAA	TGTCCTTCCCTTTCTGGATGAG	CCATTCATGAAGGCAATAGGTCTGCCC
*Fads1*	NM_146094.1	CCTTCGCGGACATTGTTTACTC	TATGGAGGTCTGCTGCTGCTAT	CTCTGGTTGGACGCTTACCTTCACCA
*Fads2*	NM_019699.1	CCCTGATCGACATTGTGAGTTC	GACGGCAGCTTCATTTATGGA	CCAGCCACAGCTCCCCAGACTTCT
*Fasn*	NM_007988	GGCATCATTGGGCACTCCTT	GCTGCAAGCACAGCCTCTCT	CCATCTGCATAGCCACAGGCAACCTC
*Hsl*	NM_010719	GAGGCCTTTGAGATGCCACT	AGATGAGCCTGGCTAGCACAG	CCATCTCACCTCCCTTGGCACACAC
*Pgc1a*	NM_008904	GACCCCAGAGTCACCAAATGA	GGCCTGCAGTTCCAGAGAGT	CCCCATTTGAGAACAAGACTATTGAGCGAACC
*Ppara*	NM_011144	TATTCGGCTGAAGCTGGTGTAC	CTGGCATTTGTTCCGGTTCT	CTGAATCTTGCAGCTCCGATCACACTTG
*Pparg1*	NM_011146	CACAATGCCATCAGGTTTGG	GCTGGTCGATATCACTGGAGATC	CCAACAGCTTCTCCTTCTCGGCCTG
*Srebp-1c*	NT_039515	GGAGCCATGGATTGCACATT	CCTGTCTCACCCCCAGCATA	CAGCTCATCAACAACCAAGACAGTGACTTCC
*36b4*	NM_007475.5	GCTCCAAGCAGATGCAGCA	CCGGATGTGAGGCAGCAG	*(SYBR Green)*
*Atgl*	NM_001163689.1	GGAGGAATGGCCTACTGAACC	ATCCTCTTCCTGGGGGACAA	
*Elovl3*	NM_007703.2	TCCATGAATTTCTCACGCGG	GCTTACCCAGTACTCCTCCAA	
*Hmox1*	NM_010442.2	AGAATGCTGAGTTCATGAAGAA	CTGCTTGTTGCGCTGTATCTC	
*Sod2*	NM_013671.3	ACAACAGGCCTTATTCCGCT	TAGTAAGCGTGCTCCCACAC	

### Statistical analysis

Statistics were performed using IBM SPSS for Windows, version 23 (IBM Corporation, Armonk, NY, USA). Time-series are plotted as median and interquartile range. Data are plotted as Tukey box-and-whisker plots and scatter plots. Analyses were carried out on all mice or samples whenever technically feasible and material was available. No data were excluded. Data were not assumed to be normally distributed, so were tested non-parametrically using the exact two-sided Mann Whitney U test. A p < 0.05 was considered statistically significant. Figures 1,2,3,4 were rendered using GraphPad Prism version 5 for Windows (GraphPad Software, La Jolla California USA, https://www.graphpad.com).

### Ethics approval

Experimental procedures were approved by an external independent animal experiment committee (CCD, Central Animal Experiments Committee, The Netherlands), and after positive advice by the Committee for Animal Experimentation of the University of Groningen. Subsequently, the study design was approved by the local Animal Welfare Body Procedures complied with the principles of good laboratory animal care following the European Directive 2010/63/EU for the use of animals for scientific purposes. This study was not performed blinded as the programming diets were visually distinct. Breeders and female offspring were terminated (CO_2_) at weaning, in compliance with the AVMA Guidelines for the Euthanasia of Animals.

## Data Availability

All data generated or analyzed during this study are included in this published article. The programming diets used in this study (see “Methods” section) are available from the corresponding author on reasonable request.

## Transcripts and proteins are provided according to the species-specific nomenclature

(T) (U/C/H) (D) CA: (Tauro) (urso/cheno/hyo) (deoxy-) cholic acid
(T) (α/β/ω)-MCA: (Tauro-) α/β/ω-muricholic acid
36B4: 60S acidic ribosomal protein P0
ACAA2: 3-Ketoacyl-CoA thiolase, mitochondrial
ACACA: Acetyl-CoA carboxylase 1
ACADS/M/VL: Short/medium/very-long-chain specific acyl-CoA dehydrogenase, mitochondrial
ACO2: Aconitate hydratase, mitochondrial
AMPK: Adenosine monophosphate-activated protein kinase
ATGL: Adipose triglyceride lipase
ATP5B: ATP synthase subunit beta, mitochondrial
BW: Body weight
CD36: Cluster of differentiation 36
CE: Cholesterol ester
cIMF: Control infant milk formula
COX5A: Cytochrome *c* oxidase subunit 5A, mitochondrial
CPT1a/2: Carnitine palmitoyltransferase 1A/2, mitochondrial
CS: Citrate synthase, mitochondrial
CXCL-1: Chemokine (C-X-C motif) ligand 1
CYCS: Cytochrome *c*, somatic
DECR1: 2,4-Dienoyl-CoA reductase, mitochondrial
DGAT1/2: Diacylglycerol *O*-acyltransferase 1/2
DLAT: Dihydrolipoyllysine-residue acetyltransferase component of pyruvate dehydrogenase complex, mitochondrial
DLD: Dihydrolipoyl dehydrogenase, mitochondrial
DLST: Dihydrolipoyllysine-residue succinyltransferase component of 2-oxoglutarate dehydrogenase complex, mitochondrial
ECHS1: Enoyl-CoA hydratase, mitochondrial
ECI1: Enoyl-CoA delta isomerase 1, mitochondrial
eIMF: Experiment infant milk formula
ELOVL3/5/6: Fatty acid elongase 3/5/6
Epi: Epididymal fat pad
ETFA/B: Electron transfer flavoprotein subunit alpha/beta, mitochondrial
ETFDH: Electron transfer flavoprotein-ubiquinone oxidoreductase, mitochondrial
FABP1: Fatty acid binding protein 1
FADS1/2: Fatty acid desaturase 1/2
FAME: Fatty acid methyl ester
FASN: Fatty acid synthase
FC: Free cholesterol
FH: Fumarate hydratase, mitochondrial
FXR: Farnesoid X receptor
GC-FID: Gas chromatography-flame ionization detector
GPX4: Phospholipid hydroperoxide glutathione peroxidase
HADH: Hydroxyacyl-coenzyme A dehydrogenase, mitochondrial
HADHA/B: Trifunctional enzyme subunit alpha/beta, mitochondrial
HMOX1: Heme oxygenase 1
HSL: Hormone sensitive lipase
IDH2: Isocitrate dehydrogenase [NADP], mitochondrial
IDH3A: Isocitrate dehydrogenase [NAD] subunit alpha, mitochondrial
IFNg: Interferon gamma
IL-1/2/5/6/10 b: Interleukin 1/2/5/6/10 (beta)
IMF: Infant milk formula
IQR: Interquartile range
LC–MS/MS: Liquid chromatography–tandem mass spectrometry
MCP-1: Monocyte chemoattractant protein 1
MDH2: Malate dehydrogenase, mitochondrial
MFGM: Milk fat globule membrane
NDUFS1: NADH-ubiquinone oxidoreductase 75 kDa subunit, mitochondrial
NEFA: Non-esterified fatty acid
NRF: Nuclear respiratory factor
OGDH: 2-Oxoglutarate dehydrogenase, mitochondrial
PDHA1: Pyruvate dehydrogenase E1 component subunit alpha, somatic form, mitochondrial
Peri: Perirenal fat pad
PGC1α: Peroxisome proliferator-activated receptor gamma coactivator 1-alpha
Phos: Phospholipids
PN: Postnatal day
PPAR-A/G1: Peroxisome proliferator-activated receptor alpha / gamma isoform 1
PRDX6: Peroxiredoxin-6
SDHA: Succinate dehydrogenase [ubiquinone] flavoprotein subunit, mitochondrial
SDHB: Succinate dehydrogenase [ubiquinone] iron-sulfur subunit, mitochondrial
SLC25A1: Tricarboxylate transport protein, mitochondrial
SLC25A11: Mitochondrial 2-oxoglutarate/malate carrier protein
SLC25A22: Mitochondrial glutamate carrier 1
SLC25A3: Phosphate carrier protein, mitochondrial
SLC25A5: ADP/ATP translocase 2
SOD2: Superoxide dismutase [Mn], mitochondrial
Srebp-1c: Sterol regulatory element-binding protein 1
SUCLA2: Succinate-CoA ligase [ADP-forming] subunit beta, mitochondrial
SUCLG1: Succinate-CoA ligase [ADP/GDP-forming] subunit alpha, mitochondrial
SUCLG2: Succinate-CoA ligase [GDP-forming] subunit beta, mitochondrial
TC: Total cholesterol
TCA cycle: Tricarboxylic acid cycle (citric acid cycle)
TG: Triglyceride
TNFα: Tumor necrosis factor alpha
UQCRC2: Cytochrome *b-c*1 complex subunit 2, mitochondrial
